# Identification of Liver and Plasma microRNAs in Chronic Hepatitis B Virus infection

**DOI:** 10.3389/fcimb.2022.790964

**Published:** 2022-06-02

**Authors:** Vladimir V. Loukachov, Karel A. van Dort, Irma Maurer, R. Bart Takkenberg, Anniki de Niet, Henk W. Reesink, Sophie B. Willemse, Neeltje A. Kootstra

**Affiliations:** ^1^ Experimental Immunology, Amsterdam University Medical Centers, University of Amsterdam, Amsterdam, Netherlands; ^2^ Amsterdam Institute for Infection and Immunity, Infectious Diseases, Amsterdam, Netherlands; ^3^ Department of Gastroenterology and Hepatology, Amsterdam University Medical Centers , University of Amsterdam, Amsterdam, Netherlands; ^4^ Department of Gastroenterology and Hepatology, Leids University Medical Center, Leiden, Netherlands

**Keywords:** biomarkers, next generation sequencing, novel therapy targets, microRNAs, hepatitis B virus

## Abstract

**Background and Aims:**

With current standard of care a functional cure for Chronic Hepatitis B (CHB) is only achieved in 1-3% of patients and therefore novel therapies are needed. Disease activity during CHB can be determined by a broad range of virological biomarkers, however these biomarkers are also targets for novel treatment strategies. The aim of this study was to identify novel miRNAs that are differentially expressed in plasma and liver in CHB, and determine whether these miRNAs may serve as biomarkers of disease stage or treatment outcome.

**Methods:**

miRNA Next-Generation-Sequencing of plasma and liver samples from CHB patient and controls was performed to identify differentially expressed miRNAs. The identified candidate miRNAs were validated by qPCR in additional plasma and liver samples from two CHB cohorts.

**Results:**

Several miRNAs in plasma and liver were found to be differentially expressed between CHB patients and controls. Of the identified miRNAs expression levels of miR-122-5p in plasma were associated with plasma HBsAg, and plasma and liver HBV-DNA levels. Expression levels of miR-223-3p, miR-144-5p and miR-133a-3p in liver were associated with plasma alanine aminotransferase levels. No correlation was observed between miRNA expression levels at baseline and treatment outcome.

**Conclusions:**

Limited overlap between plasma and liver miRNAs was found, indicating that plasma miRNAs could be useful as biomarkers for treatment outcome or viral activity during treatment. Whereas liver miRNAs are more likely to be regulated by HBV and could be potential therapeutic targets to control viral activity in liver.

## Introduction

Around one third of the global population has been infected with HBV. Worldwide around 290 million people are suffering from chronic hepatitis B (CHB), with a yearly mortality rate of 887,000 due to liver cirrhosis, liver failure and HCC. CHB can be broadly classified into two disease states which are based on the presence of either Hepatitis B e antigen (HBeAg) or hepatitis B e (HBe) antibodies in blood. HBeAg is a viral protein which is not necessary for viral replication, however it is believed that it served as an immune modulated as HBeAg presence in blood is associated with a more fulminant disease stage. Moreover patients that clear HBeAg by producing HBe antibodies have a low viral load (Tsai and Ou, 2021).

At present, only nucleos(t)ide analogues (NAs) and pegylated interferon-alpha (PEG-IFN) are registered for the treatment of CHB. NAs effectively inhibit HBV-DNA formation by blocking the conversion of the pregenomic RNA into relaxed circular DNA and therefore repressing viral replication. However, NAs do not interfere with protein production from the viral covalently closed circular (ccc)DNA, leading to ongoing production of HBsAg and HBV-RNA which can lead to the persistence of a dysfunctional HBV specific immune response. Therefore treatment with NAs rarely leads to a functional cure, defined as HBsAg loss with or without anti-HBs seroconversion plus undetectable HBV-DNA after completing a course of treatment (Cornberg et al., 2020). PEG-IFN activates innate immune pathways that interfere with viral replication, however PEG-IFN is only prescribed for a finite course of maximal 48 weeks because of severe side effects. Unfortunately, the use of PEG-IFN leads to a functional cure in around 3% of the patients (Marcellin et al., 2004) and up to 9% when used in combination with NA therapy (Takkenberg et al., 2013; Marcellin et al., 2016). However, the latter form of treatment is not standard of care.

New therapeutic options to increase the rate of functional cure are currently under development, which can be separated into two major therapeutic classes. One class consists of agents acting as immune modulators such as stimulation of RNA sensors Toll-like receptor 8 or retinoic acid-inducible gene-I (Sato et al., 2015; Amin et al., 2020). Other compounds target viral replication by degradation of HBV cccDNA reservoir using clustered regularly interspaced short palindromic repeats constructs (Schiwon et al., 2018), or target the viral mRNA transcription using RNA interference (Yuen et al., 2020). Despite all these upcoming treatment options, it is believed that complete HBV cure will be likely achieved by combining various agents with a different mechanism targeting HBV replication. Consequently, new plasma biomarkers are essential to determine hepatic HBV replication when classical virological biomarkers are no longer detectable (i.e. plasma HBsAg or HBV-DNA levels).

Several studies investigated the use of microRNAs (miRNAs) as biomarkers and targets for therapeutic interventions in various diseases, including viral hepatitis (Akamatsu et al., 2015; Tan et al., 2015; van der Ree et al., 2017). Previously, we identified plasma miRNAs that were associated with disease activity and treatment outcome in CHB patients with high viral activity (van der Ree et al., 2017). In this study we focused on identifying novel plasma and liver miRNAs that could be involved in HBV replication or could serve as important biomarkers in chronically infected patients with low viral activity.  

## Materials and Methods

### Study Population

This study was performed in pretreatment samples of CHB patients who participated in two independent investigator-initiated studies, which were described in detail previously (Takkenberg et al., 2013; de Niet et al., 2017). From the first cohort, consisting of HBeAg-negative CHB patients with HBV-DNA levels below 20,000 IU/mL, 150 plasma and 97 liver samples were available for analysis. From the second cohort, consisting of HBeAg-positive or -negative patients with HBV DNA levels >17,182 IU/mL, a total of 32 liver samples were available for analysis. Control liver tissue was obtained from 13 HBV negative patients undergoing surgical liver resection and from the resected liver tissue of the non-affected, tumor free margin surrounding the pathology was stored in RNAlater stabilizing solution (Thermo Fisher Scientific, Waltham, MA, United States) (Stelma et al., 2017). Control plasma samples were obtained from 10 healthy volunteers. Baseline characteristics of all cohorts are shown in **Table 1**.

**Table 1:** Baseline characteristics of the first CHB patient cohort (n=150), second CHB patient cohort (n=32) and liver controls (n=13).

Patient characteristics	First CHB cohort (n=150)	Second CHB cohort (n=32)	Control liver cohort (n=13)	First cohort vs. second cohort	First cohort vs. control	Second cohort vs. control
Male, n (%)	86 (57.3)	23 (71.9)	5 (39)	p = 0.1852	p = 0.3062	p = 0.0790
Age, Years, mean (SD)	43.0 (11.1)	40.1 (9.1)	59 (16)	p = 0.607	p<0.0001	p<0.0001
Region of origin:						
Caucasian, n (%)	42 (28)	11 (34.4)	10 (77)	p = 0.6126	p = 0.0009	p = 0.0236
North African, n (%)	8 (5.3)	3 (9.4)		p = 0.6438		
Central African, n (%)	37 (24.7)	6 (18.8)		p = 0.6269		
Central Asian, n (%)	6 (4)	3 (9.4)		p = 0.4099		
Southeast Asian, n (%)	19 (12.7)	9 (28.1)		p = 0.0536		
South American, n (%)	22 (14.7)		3 (23)		p = 0.6847	
ALT, U/L, median (IQR)	27 (21- 37)	103 (48-194)	30 (24-40)	p<0.0001	p>0.999	p = 0.001
**Viral characteristics**						
HbeAg positive patients, n (%)	0 (0)	13 (40.6)				
HBsAg, log10 IU/mL, mean (SD)	3.20 (0.87)	3.79 (0.94)		p = 0.0003		
HBV-DNA, log10 IU/mL, mean (SD)	2.74 (1.10)	6.61 (1.71)		p<0.0001		
HBV Genotype, n (%):						
A	29 (19.3)	11 (34.4)		p = 0.1030		
B	11 (7.3)	7 (21.9)		p = 0.0296		
C	6 (4)	2 (6.3)		p = 0.9293		
D	38 (25.3)	11 (3.1)		p = 0.4080		
E	28 (18.7)	1 (3.1)		p = 0.0555		
G	1 (0.7)					
Undeterminable	37 (24.7)					
**Liver Fibroscan and biopsy:**						
Fibroscan value (kPa), mean (SD)	5.4 (1.9)					
Fibroscan IQR range, mean (SD)	0.93 (0.88)					
Ishak fibrosis score, median (IQR)	1 (1-1)	1 (1-3)		p = 0.062		
Modified HAI score, median (IQR)	2 (2-3)	5 (3-9)		p<0.0001		
Knodell score		6 (6-10)				
Steatosis grade, median (IQR)	0 (0-1)					
cccDNA copies per hepatocyte		1 (0.33-4.42)				
% HBsAg staining, median (IQR)	15 (5-40)	30 (10-80)		p =0.002		
% HbcAg staining, median (IQR)	0 (0-0)	1 (1-10)		p<0,0001		
**Liver Pathology after resection:**						
Colorectal liver metastasis, n			4			
Hepatic adenocarcinoma, n			2			
Cholangiocarcinoma, n			2			
Hepatocellular carcinoma, n			2			
Hepatolithiasis, n			2			
Hepatic Cystadenoma, n			1			

SD, standard deviation; IQR, interquartile range; ALT, alanine transaminase; cccDNA, covalently closed circular DNA; HBcAg, hepatitis B core antigen; HBsAg, hepatitis B surface antigen; HAI, Histology Activity Index.

### Cell Lines

HepG2.2.15 cells were cultured in William’s E medium (Lonza, Switzerland) supplemented with 10% (v/v) heat-inactivated Fetal Calf Serum (FCS), 2 mM L-Glutamine, 5mM Dexamethasone, penicillin (100 U/mL) and streptomycin (100 µg/mL) at 37°C and 5% CO2. HEK293T cells were cultured in Dulbecco’s Modified Eagle Medium without Hepes (DMEM) (Lonza, Basel, Switzerland) supplemented with 10% (v/v) inactivated FCS, penicillin (100 U/ml) and streptomycin (100 mg/ml), and maintained in a humidified 10% CO2 incubator at 37°C. HepAD38 cells were cultured in Dulbecco’s Modified Eagle Medium with F-12 without Hepes (DMEM/F-12) supplemented with 10% (v/v) inactivated FCS, penicillin (100 U/ml), streptomycin (100 mg/ml) and tetracyclin (3mg/mL), and maintained in a humidified 10% CO2 incubator at 37°C.

### miRNA NGS

NGS of miRNAs was conducted at Exiqon Services, Denmark. Total RNA was isolated from 500μl plasma with proprietary RNA isolation protocol optimized for serum/plasma (no carrier added) (Exiqon Services, Denmark). Total RNA was eluted in ultra-low volume. A total of 5ul total RNA was converted into microRNA NGS libraries using the QIAseq miRNA Library Kit (Qiagen, Venlo, Netherlands). For liver samples, total RNA was isolated using the miRNeasy Micro Kit (Qiagen) and subsequently 100ng of total RNA was converted into microRNA NGS libraries. Adapters containing UMIs were ligated to the RNA and the RNA was converted to cDNA. The cDNA was amplified using PCR (22 cycles for plasma samples and 14 for liver samples) and during the PCR indices were added. The PCR samples were purified and library preparation QC was performed using either Bioanalyzer 2100 (Agilent) or TapeStation 4200 (Agilent). Based on quality of the inserts and the concentration measurements the libraries were pooled in equimolar ratios. The library pool(s) were quantified using the qPCR ExiSEQ LNA™ Quant kit (Exiqon, Denmark). The library pool was sequenced using the Illumina NextSeq500 (Illumina, San Diego, CA, United States) sequencing instrument according to the manufacturer instructions. Raw data was de-multiplexed and FASTQ files for each sample were generated using the bcl2fastq software (Illumina). FASTQ data were checked using the FastQC tool (http://www.bioinformatics.babraham.ac.uk/projects/fastqc/). Cutadapt (1.11) was used to extract information of adapter and UMI in raw reads, and output from cutadapt was used to remove adapter sequences and to collapse reads by UMI with in-house script. The reads were mapped to the reference genomes miRBase 20 and GRCh37 using Bowtie2 (2.2.2). The mapping criteria for aligning reads to abundant sequence and miRBase were that the reads have to have perfect match to the reference sequences. For mapping of the reads to the human genome one mismatch was allowed in the first 32 bases of the read. No indels were allowed in mapping. NGS data is available at the NCBI’s Gene Expression Omnibus database (www.ncbi.nlm.nih.gov/geo/, GSE162149).

### miRNA Isolation and RT qPCR

Total RNA was isolated from 200µl of plasma using the miRCURY RNA isolation kit (Exiqon, Denmark) according to the manufacturer’s protocol. Liver tissue (up to 5mg) was disrupted and homogenized for 4 x 1 minute at 30Hz using the TissueLyser II (Qiagen) and two 5mm stainless steel beads (Qiagen). Total RNA from liver samples was isolated using the miRNeasy Micro Kit, according to the manufacturer’s protocol. The RNA concentration was measured using the Nanodrop 1000 (Isogen Life Sciences, Netherlands). RNA was first polyadenylated using the E. coli Poly(A) Polymerase (M0276S, New England Biolabs, Ipswich, MA, USA) kit according to the provided protocol. The polyadenylated miRNA was reverse transcribed into cDNA using the M-MLV Reverse Transcriptase kit (Promega, Madison, WI, United States) using an Oligo-dT adapter primer (Sup. able 1), according to the protocol of the manufacturer. Individual miRNA RT-qPCR were performed using the GoTaq^®^ qPCR and RT-qPCR Systems Kit (Promega) and were run on the LightCycler^®^ 480 Instrument (Roche diagnostics, Almere, The Netherlands) using specific miRNA forward primers and an universal reverse primer (**Supplementary Table 4**). The qPCR was performed using the following program on the LightCycler: pre-incubation steps 10 min 95°C, amplification steps: 50 cycles of 10s 95°C, 20 s 58°C, 30s 72°C and a melting curve was done to confirm the purity of the PCR product and the specify of the primer.

### Production of miR-144-5p and miR-375 Overexpression Lentiviral Vectors

miR-144-5p and miR-375 sequences flanked by 100 base pair up- and downstream were cloned into a lentiviral vector in which expression is driven by the Cytomegalovirus promotor using the In-Fusion^®^ HD Cloning Kit (Takara Bio, Kusatsu, Shiga, Japan). Infectious lentiviral vector particles were produced by co-transfection of the lentiviral vector construct containing the miR-144-5p or miR-375 sequences or a GFP control (22.6 µg per construct) together with pCMV-VSV-G (8 µg), pMDLgp (14.6 µg), pRSV-Rev (5.6 µg) in HEK293T cells using calcium phosphate as previously described (Dull et al., 1998). In brief, plasmid DNA was diluted in 0.042M HEPES containing 0.15M CaCl2, subsequently mixed with an equal volume of 2× HEPES buffered saline pH 7.2, incubated at room temperature for 15 min and added to the culture medium. After 24h incubation in a humidified 3% CO2 incubator at 37°C, the culture medium was replaced and cultures were continued at 10% CO2 at 37°C. Virus was harvested at 48 and 72h after transfection and passed through a 0.22 μm filter.

### miR-144-5p and miR-375 Overexpression in HepG2.2.15 and HepAD38 Cells

HepG2.2.15 and HepAD38 cells were seeded in a 96 wells plate at a concentration of 2,000 cells per well and were transduced with either miR-144-5p, miR-375 overexpression vector, or a GFP control. Forty-eight hours after transduction the medium was replaced and HepAD38 cells were cultured in tetracyclin free medium to induce HBV replication. After six and 14 days for respectfully HepG2.2.15 and HepAD38 cells supernatant was harvested and HBV replication was analyzed by an in-house HBV-DNA PCR. In parallel, the cells viability was analyzed by a MTT assay (Sigma-Aldrich, St. Louis, Missouri, United States).

### HBV-DNA qPCR

To isolate HBV-DNA from supernatant of HepG2.2.15 and HepAD38 cells, 20 μL of supernatant was incubating with 5 μL DNase 0.05 U/μL (Promega) for 30 minutes at 37°C and subsequently the DNase was heat-inactivated for 10 minutes at 65°C. Thereafter, the supernatant was treated with 5 μL proteinase K 1 μg/mL (Sigma-Aldrich) for 30 minutes at 37°C and hereafter proteinase K was heat-inactivated for 10 minutes at 95°C. The quantification of HBV DNA was performed by GoTaq^®^ qPCR and RT-qPCR Systems Kit and were run on the LightCycler^®^ 480 Instrument using primers (PG3-forward: 5’-CAA GCC TCC AAG CTG TGC CTT G-3’, nt 1865-1886, BC1-reverse: 3’-GGA AAG AAG TCA GAA GGC AAA AAC G-5’, nt 1974-1950).

### Statistical Analyses

#### miRNA NGS

A heatmap of expression profiles was generated and a two-way hierarchical clustering analysis was performed with RStudio using trimmed mean of M-values (TMM) normalized quantifications from defined collections of samples as input. Differential expression analysis between CHB patients and controls was performed using the EdgeR statistical software package (Bioconductor, http://bioconductor.org/) in RStudio. For normalization, the TMM method based on log-fold and absolute gene-wise changes in expression levels between samples was used. Falls discovery rate (FDR) corrected p-values were calculated using the Benjamini-Hochberg method (Benjamini and Hochberg, 1995).

#### Functional Enrichment Analysis

Target-based pathway enrichment analysis of miRNAs was performed using miRWalk 3.0 (http://mirwalk.umm.uni-heidelberg.de/) with the Mirtarbase database and the 3UTR position parameters (Sticht et al., 2018). Furthermore, the targeted genes of the miRNAs were exported to DAVID (https://david.ncifcrf.gov/summary.jsp) and the functional annotation was run using only the Kyoto Encyclopedia of Genes and Genomes (KEGG) pathway parameter (Huang da et al., 2009a; Huang da et al., 2009b).

#### qPCR Candidate miRNAs

The expression of the candidate miRNAs was normalized using the 2^-ΔCt method (= 2^- (Ct miRNA of interest – arithmetic mean Ct of the two housekeeping miRNAs)). The expression level of housekeeping miRNAs was used for normalization between the input of different patients or controls of the plasma and liver samples for qPCR. The housekeeping miRNAs were selected based on the lowest stability/average TPM expression index throughout all the plasma and liver samples used for the miRNA NGS as identified by NormFinder (Andersen et al., 2004). For plasma the selected miRNAs were miR-123-3p and miR-423-3p and for liver miR-25-3p and miR-191-5p. Levels of the different miRNAs between controls and CHB patients were compared using a Mann-Whitney U test. A Benjamini-Hochberg procedure was performed in order to correct for multiple testing.

#### Baseline Characteristics

The baseline characteristics between the first CHB cohort, the second CHB cohort and liver controls were compared using either an ANOVA with Tukey’s multiple comparisons test, a Kruskal-Wallis test with Dunn’s multiple comparisons test or a chi-square test were applicable.

#### Associations with Biochemical and Virological Cohort Data

The association between miRNA expression levels and available biochemical (alanine aminotransferase (ALT)), virological (HBsAg, plasma and liver HBV-DNA and % HBsAg positive in liver), liver fibrosis or steatosis grade (Fibroscan, Ishak score, modified histology activity index (HAI) grading) analysis at baseline or therapy effect was analyzed using either Spearman correlation test or logistic regression. Before performing logistic regressions, the expression levels of miRNAs were normalized by log transformation. A Benjamini-Hochberg procedure was performed in order to correct for multiple testing. Therapy effect was defined as an absolute difference of HBsAg levels in plasma between baseline and 48 (T48) or 72 (T72) weeks or HBsAg decline of > 0.5 log between baseline and T72. Data was analyzed using Graph Pad software (version 8), RStudio (version 1.2.1335) and SPSS (version 25).

## Results

### Study Design

**Figure 1:**
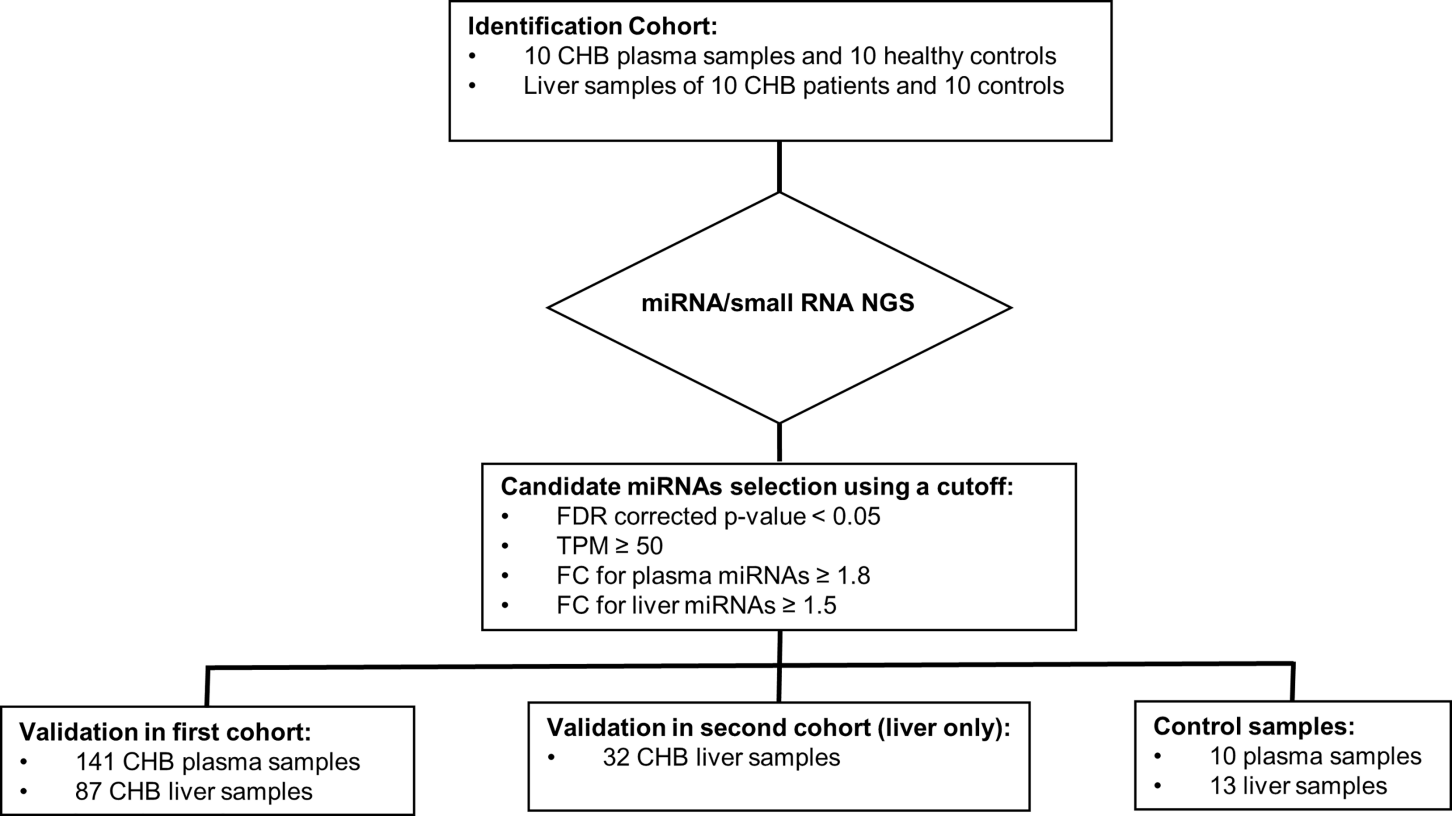
The design of the study. The current study was performed in pretreatment plasma and liver tissue samples obtained from CHB patients participating in two independent CHB cohorts (Cohort 1: HBeAg-negative CHB patients with HBV DNA levels <20,000 IU/mL; Cohort 2: HBeAg-positive or -negative CHB patients with HBV DNA levels >17,182 IU/mL) and comprised two steps: the identification and the validation step. In the identification step, HBV associated miRNAs were identified in matching plasma and liver tissue samples of an identification cohort consisting of 10 CHB patients (selected from the first CHB cohort) and 10 control samples by miRNA/small RNA NGS. In the validation step, candidate miRNAs were validated by qPCR in two confirmation cohorts that consisted of the remaining plasma (n=141) and liver samples (n=87) of the patients of the first cohort, liver samples of the second cohort (n=32), and control plasma (n=10) and liver tissue samples (n=13).

The current study was performed in pretreatment plasma and liver tissue samples obtained from two independent CHB cohorts (Cohort 1: HBeAg-negative CHB patients with HBV DNA levels <20,000 IU/mL (de Niet et al., 2017); Cohort 2: HBeAg-positive or -negative CHB patients with HBV DNA levels >17,182 IU/mL (Takkenberg et al., 2013), and comprised two steps (**Figure 1**): the identification and the validation step. In the identification step, HBV associated miRNAs were identified in matching plasma and liver tissue samples of an identification cohort consisting of 10 CHB patients and 10 control samples by miRNA/small RNA NGS which were selected from the first CHB cohort. We selected 5 male and 5 female CHB patients of whom 5 patients were infected with HBV genotype A virus and 5 patients with HBV genotype D virus. In the validation step, candidate miRNAs were validated in two confirmation cohorts that consisted of the remaining plasma and liver samples of the patients of the first cohort and 32 liver samples of the second cohort by qPCR. The expression of candidate miRNAs was compared to the 10 control plasma samples and 13 control liver tissue samples.

### Identification of miRNAs Differentially Expressed in Liver and Plasma of CHB Patients Using miRNA/Small RNA NGS

**Figure 2:**
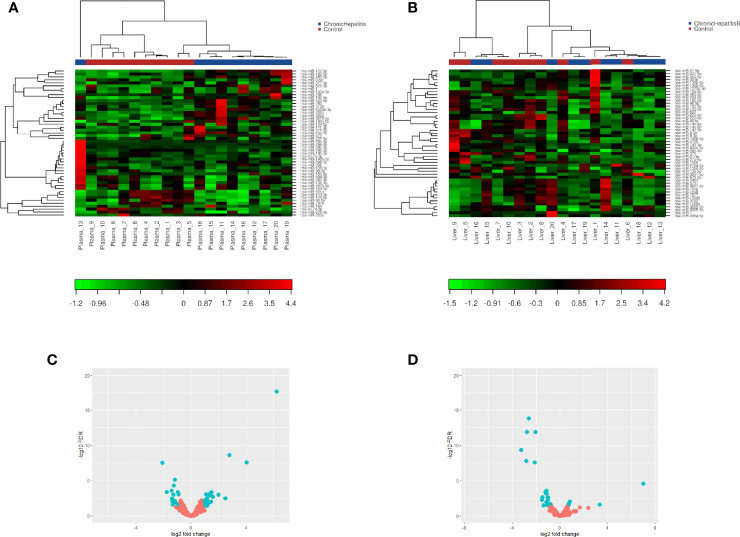
Two-way hierarchical clustering analysis and volcano plot of identified plasma and liver tissue miRNA. miRNA/small RNA NGS was performed using total RNA isolated from matching plasma (n=10) and liver biopsies(n=10) of the identification cohort. A heatmap of plasma **(A)** or liver **(B)** expression profiles was generated and a two-way hierarchical clustering analysis was performed using TMM normalized quantifications from defined collections of samples as input. Red represents an expression level above the mean and green below the mean. Volcano plot of differentially expressed miRNAs between CHB patients and controls in plasma **(C)** and liver tissue **(D)** samples. miRNAs that pass the cut-off criteria (FDR ≤ 0.05 and FC ± 1.8 for plasma or FC ± 1.5 for liver) are shown in blue.

miRNA/small RNA NGS was performed using total RNA isolated from matching plasma and liver biopsies of the identification cohort. On average 25 million raw reads were obtained for each plasma sample, of which 4.3 million reads remained after correction for length and quality. The average mapping rate to the miRNA reference database was 65.7%. For liver samples, on average 21 million raw reads were obtained for each sample, of which 12.7 million reads remained after correction for length and quality. The average mapping rate to the miRNA reference database was 47.1%. After mapping, all miRNAs with less than 5 counts per million per group were excluded from further analysis. The heterogeneity between groups was assessed, and based on the hierarchical cluster plot and low read counts (<500,000 reads) one CHB plasma sample (nr 13), 3 control plasma samples (nr 1, 4, and 6), and 3 CHB liver sample (nr 15, 16 and 19) were excluded from further analysis (**Figures 2A, B**). Candidate miRNAs were selected using a cutoff falls discovery rate corrected p-value of 0.05, a fold change of 1.5 for liver and 1.8 for plasma samples. A total of 45 and 30 miRNAs were found to be differentially expressed between CHB patients and controls in plasma and liver, respectively (**Figures 2C, D**). For further selection of miRNAs to be validated we applied a filter of a minimal TPM of 50. This resulted in 23 miRNAs for the plasma samples of which 12 were upregulated and 11 downregulated, and a total of 18 miRNA in the liver samples, of which 3 were upregulated and 15 downregulated (**Tables 2**, **3**). Furthermore, 5 of these miRNAs (miR-451a, miR-182-5p, miR-144-3p, miR-144-5p and miR-206) were differentially expressed in both plasma and liver samples.

**Table 2:** Differentially expressed miRNAs between CHB patients and controls in plasma.

Names	log FC	log CPM	p value	FDR
hsa-miR-122-5p	2.799267713	14.42359219	1.36E-11	2.40E-09
hsa-miR-144-5p	-2.085790977	7.390978459	3.52E-10	3.10E-08
hsa-miR-7-5p	-1.164231562	8.604354064	1.05E-07	7.42E-06
hsa-miR-190a-5p	-1.250280028	8.467641072	8.28E-07	4.87E-05
hsa-miR-182-5p	-1.399547709	9.556505395	5.14E-06	0.000259043
hsa-miR-454-3p	-0.954149239	9.589666605	1.01E-05	0.000417074
hsa-miR-144-3p	-1.757703758	8.998973012	1.13E-05	0.000417074
hsa-miR-4433b-3p	1.427556917	5.934071689	2.30E-05	0.00073653
hsa-miR-423-5p	1.310898419	12.36965441	3.35E-05	0.000844194
hsa-miR-125a-5p	-0.90941848	11.36467465	3.93E-05	0.000923984
hsa-miR-99b-5p	-1.26565281	8.872803688	4.97E-05	0.001031627
hsa-miR-206	2.489188961	5.745344907	0.000194778	0.003274126
hsa-miR-183-5p	-1.385567083	7.794076129	0.000244106	0.003909345
hsa-miR-146a-5p	0.902914756	15.11570602	0.000489973	0.006888997
hsa-let-7e-5p	-1.165237237	10.78400728	0.000645259	0.007953655
hsa-miR-451a	-1.362727954	12.13869939	0.000768813	0.009046365
hsa-miR-483-5p	1.451908189	5.881725394	0.000888296	0.009881216
hsa-miR-320a	0.958539865	10.71012277	0.001180799	0.011265459
hsa-miR-584-5p	0.896577295	11.29900783	0.001261937	0.011722734
hsa-miR-335-5p	0.862608153	10.28986294	0.00170718	0.013100749
hsa-miR-224-5p	1.112302319	6.767858863	0.002486796	0.017556781
hsa-miR-625-3p	1.052592088	9.183494765	0.00276667	0.019149696
hsa-miR-215-5p	1.175501944	5.965196141	0.006478616	0.036300816

FC, fold change; CPM, counts per million; FDR, false discovery rate corrected p-value.

**Table 3:** Differentially expressed miRNAs between CHB patients and controls in liver.

Names	log FC	log CPM	p value	FDR
hsa-miR-199b-5p	-2.6078	7.507131	4.85E-17	1.51E-14
hsa-miR-21-3p	-2.06604	6.510362	7.89E-15	1.23E-12
hsa-miR-223-3p	-2.7658	11.77646	1.30E-14	1.35E-12
hsa-miR-10b-5p	-2.83762	5.458021	2.67E-10	1.67E-08
hsa-miR-206	7.019629	5.6105	6.53E-07	2.91E-05
hsa-miR-338-3p	-1.19515	6.762663	9.37E-06	0.000325
hsa-miR-182-5p	-1.09799	5.311133	2.52E-05	0.000716
hsa-miR-9-5p	-1.48441	5.339281	8.55E-05	0.002224
hsa-miR-143-3p	-1.03774	13.90181	0.000112	0.002499
hsa-miR-451a	-1.1396	9.089024	0.000599	0.010381
hsa-miR-143-5p	-0.83687	6.976719	0.001237	0.019294
hsa-miR-144-5p	-1.0745	7.712035	0.001758	0.024926
hsa-miR-133a-3p	3.375883	6.370239	0.001721	0.024926
hsa-miR-331-3p	0.730901	5.750636	0.002112	0.026357
hsa-miR-375	-1.06235	6.937652	0.002379	0.028551
hsa-miR-141-3p	-1.38884	6.432368	0.0029	0.032311
hsa-miR-145-3p	-0.81099	5.944412	0.003285	0.03534
hsa-miR-144-3p	-0.94376	7.88644	0.003612	0.03757

FC, fold change; CPM, counts per million; FDR, false discovery rate corrected p-value.

### Target-Based Pathway Enrichment Analysis

The differentially expressed plasma and liver miRNAs were subjected to target-based pathway enrichment analysis using miRWalk 3.0 and DAVID. Several different KEGG pathways were identified when correction for FDR was performed (**Supplementary Tables 2–5**). One of the most significant pathways were related to HBV infection. Next to infectious diseases, a substantial amount of these pathways were related to oncogenic diseases like pancreatic cancer or colorectal cancer. Moreover, several miRNAs targeted genes involved in the hepatocellular carcinoma pathway.

### Validation of the Identified Differentially Expressed of miRNAs in Liver and Plasma of CHB Patients in the First Conformation Cohort

**Figure 3:**
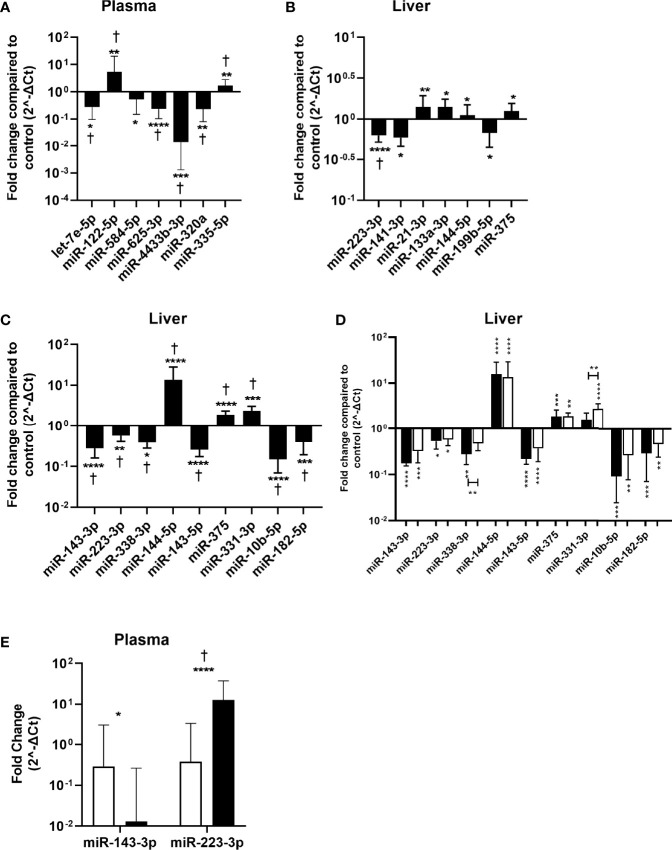
Validation of identified miRNAs in plasma and liver tissue samples. miRNA expression in plasma and liver tissue was determined by RT qPCR and was compared between the CHB patients and controls. The fold change in normalized miRNA expression levels in plasma samples (n=141) of the first conformation cohort **(A)**, liver tissue samples of the first (n=87) **(B)** or second (n=32) **(C)** conformation cohort as compared to controls (plasma n=10, liver n=13) are shown. **(D)** The fold change of normalized miRNA expression levels in liver tissue from HBeAg positive (black bars) and HBeAg negative (open bars) patients from the second cohort as compared to controls are shown. **(E)** Differential expression of confirmed CHB associated liver miRNAs in plasma. Open bars represent the control and black bars the CHB patients. Data is presented as median and interquartile range. Differences between the groups were determined by Mann Whitney U test and only significant differently expressed miRNAs are shown. * p<0.05; ** p<0.01; *** p<0.001; **** p<0.0001. For panel **(A–C)** ^†^represents p<0.05 after correction for multiple testing.

Next, these miRNAs were validated in the remaining pre-treatment 141 plasma and 87 liver samples of CHB patients by performing RT-qPCRs. Seven out of 23 miRNAs were found to be differentially expressed between CHB patients and controls in plasma. miRNAs let-7e, miR-625-3p, miR-4433b-3p, miR-584-5p and miR-320a were found to be downregulated, and miR-122-5p and miR-335-5p were upregulated in plasma of CHB patients (**Figure 3A**). The specific detection of nine miRNAs failed due to low expression levels or primer aspecificity as was observed by melting curve analysis (miR-7-5p, miR-144-3p, miR-182-5p, miR-183-5p, miR-190a-5p, mir-206, miR-215-5p, miR-224-5p, miR-454-3p). For liver, seven out of the 18 miRNAs were found to be differentially expressed between CHB patients and controls. miRNAs miR-21-3p, miR-133a-3p, miR-144-5p and miR-375 were upregulated and miR-223-3p, miR-141-3p, miR-199b-5p were downregulated in CHB patients (**Figure 3B**). For three miRNAs the specific detection using qPCR failed due to low expression levels or primer aspecificity as was observed by analyses of the melting curves (miR-9-5p, miR-206 and miR-145-3p).

### Validation of Candidate miRNA in Liver of the Second Conformation Cohort

The identified miRNAs in liver were also validated in a second cohort consisting of 32 pre-treatment liver tissue samples from CHB patients. Nine out of the 18 miRNAs were found to be differentially expressed between CHB patients and controls. miRNAs miR-331-3p, miR-144-5p and miR-375 were upregulated and miR-143-3p, miR-223-3p, miR-10b-5p, miR-182-5p, miR-143-5p and miR-338-3p were downregulated (**Figure 3C**). For six miRNAs the specific detection using qPCR failed due to low expression levels or primer aspecificity as was observed by melting curve analysis (miR-451a, miR-199b-5p, miR-141-3p, miR-9-5p, miR-206 and miR-145-3p). The cohort consisted of both HBeAg positive and HBeAg negative patients. When analyzing these groups separately we observed that miR-144-5p and miR-375 were upregulated, and miR-143-3p, miR-223-3p, miR-10b-5p, miR-182-5p and miR-143-5p were downregulated in both the HBeAg negative and positive groups compared to controls. Upregulation of miR-331-3p was only observed in the HBeAg negative group and miR-338-3p was only downregulated in the HBeAg positive group compared to control (**Figure 3D**).

### Validation of Liver miRNAs Expression in Plasma

None of the validated differentially expressed miRNA in liver were also identified in plasma and vice versa. To determine whether the differentially expressed liver miRNAs could serve as biomarkers in plasma, we measured the expression levels of validated liver miRNA in plasma. We found that miR-143-3p was downregulated and miR-223-3p was upregulated in plasma of CHB patients (**Figure 3E**).

### Association of Plasma or Liver miRNA Levels and Baseline Characteristics of CHB Patients

To determine whether the expression levels of the validated miRNAs were associated with various baseline characteristics in our cohorts, correlation analyses were performed. For both cohorts, baseline characteristic included liver damage (plasma ALT levels, degree of fibrosis and/or steatosis in the liver biopsy) and viral replication markers (plasma levels of HBsAg or plasma and liver HBV-DNA levels, or percentage of HBsAg positively stained cells in the liver biopsy). For the miRNAs identified in plasma, we found that the expression of miR-122-5p was positively associated with plasma HBsAg levels and with plasma and liver HBV-DNA levels. For miR-320a and miR-625-3p, a positively association was observed with plasma HBV-DNA levels. In liver samples from the first cohort, we found that miR-223-3p, miR-144-5p and miR-133a-3p were negatively associated with ALT levels (**Table 4**). In the liver samples from the second cohort no correlations were observed.

**Table 4:** The association of plasma and liver miRNAs levels with biomarkers of HBV replication and liver disease.

	HBV replication^#^				Liver disease^#^				
	**ALT**	**HBsAg**	**Plasma HBV-DNA**	**Liver HBV-DNA**	**Liver Stiffness**	**Modified HAI Grading**	**Steatosis grade**	**% HBsAg staining**	**Ishak fibrosis score**
Plasma									
let-7e-5p	0.169	-0.124	0.131	0.114	0.057	-0.017	0.191	0.093	0.205
miR-122-5p	0.222	0.343 ****	0.244 *	0.396 **	0.184	0.072	0.108	0.188	0.219
miR-320a	0.072	-0.081	0.258 *	-0.061	0.055	0.041	0.025	-0.144	0.147
miR-335-5p	-0.026	0.028	0.102	0.149	0.041	0.256	0.114	-0.048	0.151
miR-584-5p	-0.039	-0.026	0.042	-0.042	-0.038	0.052	-0.023	-0.129	0.080
miR-625-3p	0.110	-0.028	0.264 *	-0.062	0.007	-0.007	0.105	-0.062	0.068
miR-4433b-3p	-0.035	0.065	0.136	0.183	0.148	-0.213	0.042	-0.029	0.120
									
Liver									
miR-223-3p	-0.252 *	0.140	-0.217	0.087	-0.039	-0.106	0.006	0.041	0.079
miR-144-5p	-0.253 *	0.027	-0.186	0.163	-0.125	-0.207	-0.113	0.037	-0.070
miR-199b-5p	-0.075	-0.006	-0.186	0.251	0.151	-0.155	0.025	0.150	0.222
miR-375	-0.102	0.002	0.004	0.039	-0.096	-0.093	0.120	-0.058	0.053
miR-141-3p	0.021	0.254	-0.193	-0.073	0.254	0.031	0.258	-0.195	0.204
miR-21-3p	-0.137	0.177	-0.021	0.025	-0.042	0.032	0.151	0.047	-0.010
miR-133a-3p	-0.337 **	0.043	-0.118	0.063	-0.072	-0.230	-0.032	-0.012	0.032

**^#^** Spearman correlation coefficient. *: p<0.05, **: p<0.01, ****: p<0.0001. HBsAg, hepatitis B surface antigen; ALT, alanine transaminase; HAI, Histology Activity Index.

### Associations Between miRNA Levels in Plasma and the Effect of peg-IFN and NAs Therapy in the First Cohort

Next we assessed whether miRNAs expression levels in plasma at baseline of the first cohort were associated with the effect of treatment (48 weeks of peg-IFN and either adefovir or tenofovir) as determined by either the decline of absolute HBsAg levels or HBsAg decline of > 0.5 log between baseline and 48 weeks or 72 weeks. These parameters were chosen as in this cohort functional cure was only found in approximately 4% of the patients. No correlation between the expression levels of the different miRNAs and absolute HBsAg levels decline in plasma (**Table 5**). However, a relationship between the expression levels of miR-122-5p and HBsAg decline of > 0.5 log between baseline and 72 weeks using univariate logistic regression (odds ratio 0.446, p = 0.025) was observed, although this association was lost after correction for multiple testing (p = 0.175).

**Table 5:** The association between baseline plasma miRNAs levels and HBV-DNA decline after 48 and 72 weeks in treated and non-treated CHB.

miRNA	Decline HBsAg T48^#^		Decline HBsAg T72^#^	
	Treatment	No Treatment	Treatment	No Treatment
let-7e-5p	-0.020	0.169	0.012	0.088
miR-122-5p	0.199	-0.375	0.241	0.000
miR-320a	0.244	0.164	0.064	0.362
miR-335-5p	0.087	-0.218	0.086	0.056
miR-584-5p	0.034	-0.133	-0.087	0.055
miR-625-3p	-0.043	0.067	-0.141	0.133
miR-4433b-3p	0.192	0.125	0.122	-0.022

^#^Spearman correlation coefficient. HBsAg, hepatitis B surface antigen. T48, 48 weeks post treatment; T72, 72 weeks post treatment.

### The Effect of miR-144-5p and miR-375 on HBV Replication

**Figure 4:**
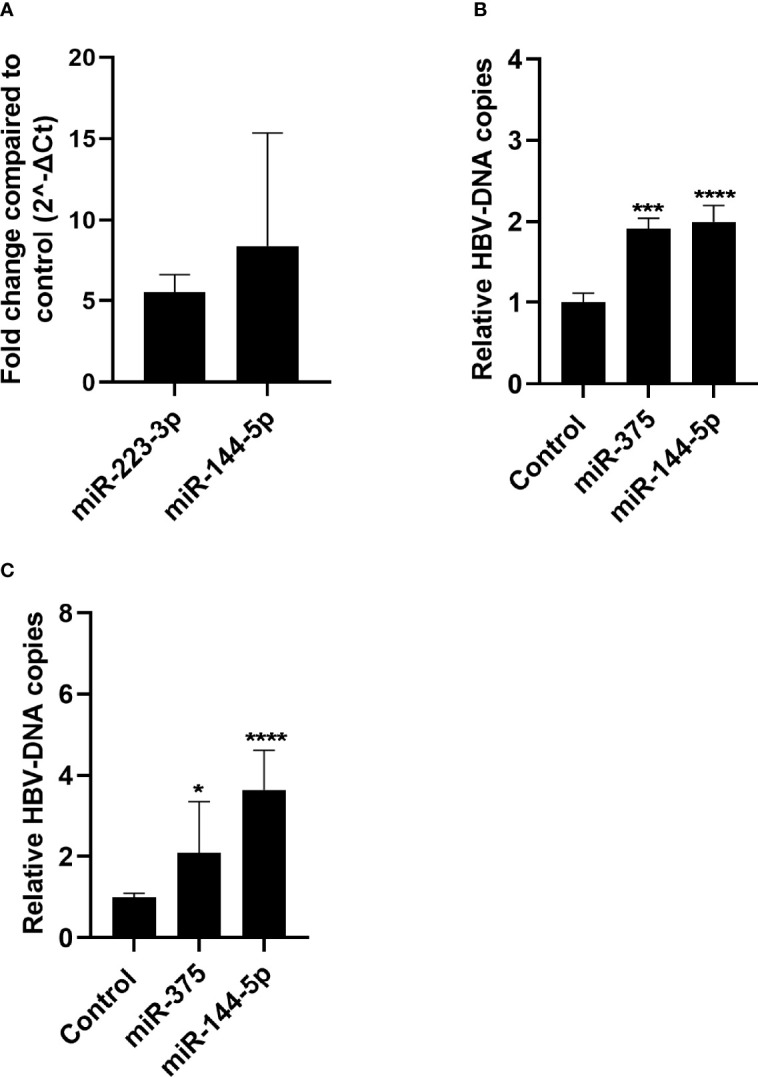
Effect of miR-144-5p and miR-375 overexpression on HBV replication *in vitro*. miR-144-5p and miR-375 were overexpressed using a lentiviral vector in HepG2.2.15 **(A)** and HepAD38 **(B)** cells. HBV-DNA levels were measured in the supernatant. HBV-DNA levels shown were corrected for cell viability analysed by MTT assay. **(C)** Overexpression of miR-144-5p and miR-375 by lentiviral transduction in HepG2.2.15 cells was determined by qPCR and is expressed as fold increase as compared to the GFP control. Data is presented as median and interquartile range. Differences between the groups were determined by Mann Whitney U test. *: p<0.05, ***: p<0.001, ****: p<0.0001.

As miR-144-5p and miR-375 were both upregulated in the liver of CHB patients, the effect of overexpression of these miRNAs on HBV replication was investigated in HepG2.2.15 and HepAD38 cells using a lentiviral overexpression vector. In both cell lines HBV DNA levels were increased as compared to control in the supernatant after miR-144-5p and miR-375 overexpression (**Figure 4**).

## Discussion

After performing the initial NGS identification screen, a targeted gene pathway enrichment analysis identified several pathways involving candidate miRNAs from both plasma and liver. One of the most significant targeted gene enrichment pathways was related to HBV infection, which is an indicator that the identified miRNAs could be involved in HBV replication. Next to infectious diseases a substantial amount of the identified pathways were related to oncogenic diseases, including hepatocellular carcinoma. As CHB is strongly associated with the development of hepatocellular carcinoma, this supports the suggestion that these miRNAs are involved in HBV replication. Interestingly, our miRNA screen showed only limited overlap of differentially expressed miRNAs in both plasma and liver samples of CHB patients. This indicates that liver and plasma miRNAs in CHB are representative of different entities. Differential expression of miRNA in liver might be caused by ongoing local inflammation and immune responses as well as the regulation of miRNA expression by the virus itself. Plasma miRNAs originate from various cellular and tissue sources. Differential secretion of miRNAs for instance by activated immune cells or infected hepatocytes may be reflected in plasma whereas this may not be detectable in liver tissue.

After validation, we found that miRNAs let-7e, miR-625-3p, miR-4433b-3p, miR-584-5p and miR-320a were downregulated, and miR-122-5p and miR-335-5p were upregulated in plasma of untreated CHB patients. Since CHB patients with a low viral load currently do not receive therapy, we subsequently investigated whether baseline miRNA expression levels in plasma could be used as biomarkers to identify a subset of patients that potentially could benefit from therapy. We found that the expression of miR-122-5p was positively associated with baseline HBsAg and plasma and liver HBV-DNA levels. However, no correlation of miR-122-5p with either HBsAg or HBsAg decline after treatment with peg-IFN and either adefovir or tenofovir was observed. As miR-122-5p is the most commonly expressed miRNA in the liver, the observed association at baseline could be the result of liver damage cause by immune mediated kill of HBV infected hepatocytes. In addition, we noticed a trend between the expression levels miR-122-5p and ALT levels which strengthen this hypothesis. In contrast to the current study, it was previously shown that plasma miR-122-5p expression levels were associated with a virological response in CHB after NA treatment (Wu et al., 2019). However, this study lacked an essential untreated control group, as another study showed that plasma miR-122-5p is an independent predictor of HBsAg seroclearance in patients who had not received therapy (Akuta et al., 2018). For miR-320a and miR-625-3p a positive association with baseline plasma HBV-DNA levels was observed, however no direct relationship between these miRNAs and HBV has been described. miR-335-5p was previously associated with CHB, and the expression levels of miR-335-5p were found to be predictive for HBsAg clearance in patients having a low viral load treated with PEG-IFN (Yang et al., 2018). However, we could not confirm this observation in our present study. For the other identified plasma miRNAs no association with CHB has previously been described.

In liver samples from both cohorts, miR-144-5p and miR-375 are upregulated and miR-223-3p is downregulated in CHB patients. Overexpression of both miR-144-5p and miR-375 in HepG2.2.15 and HepAD38 resulted in increased levels of HBV-DNA in the supernatant. This indicates that upregulation of miR-144-5p and miR-375 in liver can support HBV replication and persistence in CHB, making these miRNAs interesting therapeutic targets.

In the first cohort liver expression of miR-21-3p and miR-133a-3p was upregulated whereas miR-141-3p and miR-199b-5p were downregulated. However, this could not be confirmed in liver tissue obtained from the second cohort. Where miR-331-3p was upregulated and miR-143-3p, miR-10b-5p, miR-182-5p, miR-143-5p and miR-338-3p were downregulated. This discrepancy may be explained by the patient selection in our cohorts, while the first cohort consists of HBeAg negative patients with low viral load, the second cohort included HBeAg positive and negative patients with high viral load and more liver damage. Therefore, we believe that these miRNAs are not reflecting HBV infection itself, but are related to variation between the CHB patient groups like the level of inflammation and immune responses related to HBV activity or liver damage.

Of the identified liver miRNAs only miR-141-3p was described to be involved in HBV replication. *In vitro* studies show that miR-141-3p inhibited HBV replication by either targeting peroxisome proliferator-activated receptor (PPAR)-alpha resulting in reduced HBV promoter activity, or downregulate Sirtuin 1 and subsequently autophagy-mediated HBV inhibition (Hu et al., 2012; Yang et al., 2017).

The second CHB cohort consisted of both HBeAg negative and positive patients, and this allowed us to analyze the HBeAg positive and HBeAg negative groups separately. Discrepancies between the HBeAg positive and negative groups were only observed for miR-331-3p and miR-338-3p. Upregulation of miR-331-3p was observed in the HBeAg negative group when compared to the controls, but no significant upregulation of this miRNA was observed in the first cohort with only HBeAg negative patients with low viral load.

The five differentially expressed miRNAs in both plasma and liver tissue of the identification cohort could not be validated in the validation cohorts. However when the expression of the identified liver miRNAs were analyzed in plasma samples, we found that the expression levels of miR-223-3p and miR-143-3p differed between CHB patients and controls in both plasma and liver. Surprisingly, miR-223-3p was upregulated in plasma of CHB patients whereas it was downregulated in both liver samples cohorts. Increased levels of miR-223-3p in serum of CHB patients has previously been reported (Xu et al., 2011; Bao et al., 2017), conforming our results in plasma. Although the low expression of miR-223-3p in liver of CHB patients could be explained by the downregulation of miR-223-3p by HBx (Yu et al., 2016), the observed increased levels of miR-223-3p in plasma may result from active shuttling of this miRNAs from the liver by for instance packaging into HBsAg particles as suggested previously (Novellino et al., 2012). Moreover, miR-223 has immune modulatory functions and has been shown to be involved in a variety of immune modulated diseases by the regulation of the NFκB pathway or the NLRP3 inflammasome, making this miRNA an interesting target for further investigation in CHB (Valmiki et al., 2019; Xu et al., 2020). Indeed, the expression levels of miR-143-3p in peripheral blood mononuclear was identified as a biomarker in patients with hepatitis B-related acute-on-chronic liver failure (Ding et al., 2015).

Previously, several other studies have been performed to identify the miRNAs expression profile of plasma samples (Akamatsu et al., 2015; van der Ree et al., 2017) or liver (Liu et al., 2009; Nielsen et al., 2018; Singh et al., 2018) in CHB patients. However, hardly any overlap between plasma and liver miRNAs identified in the various studies was observed. Only the expression of miR-122-5p was found to be upregulated in plasma of CHB patients and was confirmed in an additional study (Ma et al., 2020). The discrepancy for the other miRNAs could be explained by several reasons. First, in our study we used miRNA NGS while previous studies mostly used universal RT microRNA PCR or a miRNA microarray techniques to identify the miRNAs. Secondly, patients included in the studies differed in their CHB disease stages, making a direct comparison to our current study difficult. Of the studies performed in liver, one study focused on differential expression of miRNAs in relation to the different stages of infection and liver fibrosis (Singh et al., 2018), whereas the remaining other two studies analyzed miRNA expression in *in vitro* infection models using HepG2 and the HepG2.2.15 cell lines (Liu et al., 2009; Nielsen et al., 2018).

In conclusion, in this study several novel miRNAs were identified that were differentially expressed in either plasma or liver samples of CHB patients. Of which overexpression of miR-144-5p and miR-375 *in vitro* (HepG2.2.15 and HepAD38) resulted in an increase in HBV. The expression of miR-122-5p in plasma was positively associated with baseline HBsAg in plasma and HBV-DNA in plasma and liver. Baseline expression levels of plasma miR-320a and miR-625-3p were associated with baseline plasma HBV-DNA levels. Liver baseline expression levels of miR-223-3p, miR-144-5p and miR-133a-3p were associated with plasma ALT levels which could be an indication that these miRNAs are involved in liver injury. In our study we found limited overlap between the identified plasma and liver miRNAs, indicating that plasma miRNAs could be useful as biomarkers for treatment outcome or viral activity during treatment especially in the context of novel treatment regimens which target viral transcription or viral proteins. Whereas, liver miRNAs may regulated HBV replication and could be potential therapeutic targets to control viral replication in the liver. 

## Data Availability Statement

The datasets presented in this study can be found in online repositories. The names of the repository/repositories and accession number(s) can be found below: https://www.ncbi.nlm.nih.gov/geo/, GSE162149.

## Ethics Statement

The studies involving human participants were reviewed and approved by Amsterdam University Medical Centers, location University of Amsterdam. The patients/participants provided their written informed consent to participate in this study.

## Author Contributions

Conceptualization; NK and VL; Data curation: KD, VL, and IM; Formal analysis: VL; Funding acquisition: NK and HR; Investigation: VL, KD, and IM; Methodology: NK and VL; Project administration: NK and VL; Resources: NK, AN, SW, and BT; Supervision: NK; Validation: NK and VL; Visualization: VL; Writing original draft: NK and VL; Writing: review & editing NK, VL, AN, HR, SW, and RBT. All authors contributed to the article and approved the submitted version.

## Funding

This study was funded by the Dutch Research Council Open Technology Program (project number 15783).

## Conflict of Interest

The authors declare that the research was conducted in the absence of any commercial or financial relationships that could be construed as a potential conflict of interest.

## Publisher’s Note

All claims expressed in this article are solely those of the authors and do not necessarily represent those of their affiliated organizations, or those of the publisher, the editors and the reviewers. Any product that may be evaluated in this article, or claim that may be made by its manufacturer, is not guaranteed or endorsed by the publisher.
