# Supplementary material for: Identification of Liver and Plasma microRNAs in Chronic Hepatitis B Virus infection
Source: Front Cell Infect Microbiol. 2022 Jun 2;12:790964. doi: 10.3389/fcimb.2022.790964 (PMC9201251; doi:10.3389/fcimb.2022.790964)
Supplement: Supplementary file 1 [file Table_1.docx]

Supplementary Material

Supplementary table 1. Primers used for the qPCR.

| miRNA | Sequence |
| --- | --- |
| Oligo-dT adapter primer | 5'-CATAGACCTGAATGGCGGTAAGGGTGTGGTAGGCGAGACA  TTTTTTTTTTTTTTTTTTTT-3' |
| Universal reverse primer | 5'-GCATAGACCTGAATGGCGGTA-3' |
| miR-146a-5p_Forward | 5'-TGAGAACTGAATTCCATGGGTTA-3' |
| miR-423-5p_Forward | 5'-GGGCAGAGAGCGAGACTTTA-3' |
| miR-451a_Forward | 5'-CGAAAACCGTTACCATTACTGA-3' |
| miR-584-5p_Forward | 5'-TTATGGTTTGCCTGGGACTG-3' |
| miR-125a-5p_Forward | 5'-GAGACCCTTTAACCTGTGAAAAA-3' |
| miR-320a_Forward | 5'-CTGGGTTGAGAGGGCGAAA-3' |
| miR-335-5p_Forward | 5'-TCAAGAGCAATAACGAAAAATGTAA-3' |
| miR-454-3p_Forward | 5'-TAGTGCAATATTGCTTATAGGGT-3' |
| miR-182-5p_Forward | 5'-GCTTTGGCAATGGTAGAACTCA-3' |
| miR-625-3p_Forward | 5'-AGAACTTTCCCCCTCAAAAA-3' |
| miR-144-3p_Forward | 5'-CGCCTACAGTATAGATGATGTACTAAA-3' |
| miR-99b-5p_Forward | 5'-CACCCGTAGAACCGACCTTG-3' |
| miR-7-5p_Forward | 5'-CGTGGAAGACTAGTGATTTTGTTG-3' |
| miR-190a-5p_Forward | 5'-CGCATGATATGTTTGATATATTAGGTA-3' |
| miR-183-5p_Forward | 5'-CGCCTATGGCACTGGTAGAA-3' |
| miR-144-5p_Forward | 5'-CGGATATCATCATATACTGTAAGAAAAA-3' |
| miR-224-5p_Forward | 5'-GCAAGTCACTAGTGGTTCCGTTA-3' |
| miR-215-5p_Forward | 5'-CGCATGACCTATGAATTGACAGAC-3' |
| miR-4433b-3p_Forward | 5'-GAGTGGGGGGTGGGACGTAAA-3' |
| miR-483-5p_Forward | 5'-ACGGGAGGAAAGAAGGGAGA-3' |
| miR-206_Forward | 5'-TGGAATGTAAGGAAGTGTGTGGA-3' |
| miR-143-3p_Forward | 5'-TGAGATGAAGCACTGTAGCTCAAA-3' |
| miR-223-3p_Forward | 5'-TGTCAGTTTGTCAAATACCCCAAA-3' |
| miR-199b-5p_Forward | 5'-CCCAGTGTTTAGACTATCTGTTCAA-3' |
| miR-143-5p_Forward | 5'-CAGTGCTGCATCTCTGGTAAA-3' |
| miR-375_Forward | 5'-GTTCGGCTCGCGTGAAA-3' |
| miR-338-3p_Forward | 5'-TCCAGCATCAGTGATTTTGTTG-3' |
| miR-141-3p_Forward | 5'-CGTAACACTGTCTGGTAAAGATGGA-3' |
| miR-21-3p_Forward | 5'-ACCAGTCGATGGGCTGTAAA-3' |
| miR-145-3p_Forward | 5'-GGATTCCTGGAAATACTGTTCTAAA-3' |
| miR-133a-3p_Forward | 5'-CTTTGGTCCCCTTCAACCAG-3' |
| miR-331-3p_Forward | 5'-CCCTGGGCCTATCCTAGAAA-3' |
| miR-9-5p_Forward | 5'-CGCTCTTTGGTTATCTAGCTGTATG-3' |
| miR-128-3p_Forward | 5'-CCGCACTCACAGTGAACC-3' |
| miR-423-3p_Forward | 5'-TGAGGCCCCTCAGTAAAAA-3' |
| miR-25-3p_Forward | 5'-CATTGCACTTGTCTCGGTCTG-3' |
| miR-191-5p_Forward | 5'-AACGGAATCCCAAAAGCAG-3' |
| miR-122-5p_Forward | 5'-TGGAGTGTGACAATGGTGTTTG-3' |
| let-7e-5p_Forward | 5'-CGTGAGGTAGGAGGTTGTATAGTT-3' |
| miR-10b-5p_Forward | 5'-TACCCTGTAGAACCGAATTTGTG-3' |

**Supplementary table 2. Identified KEGG pathways of the differentially expressed plasma miRNAs subjected to a target-based pathway enrichment analysis using miRWalk 3.0.**

| Pathway | P value | FDR |
| --- | --- | --- |
| hsa05219 Bladder cancer | <0.0000 | <0.0000 |
| hsa04722 Neurotrophin signaling pathway | <0.0000 | <0.0000 |
| hsa05212 Pancreatic cancer | <0.0000 | <0.0000 |
| hsa05165 Human papillomavirus infection | <0.0000 | <0.0000 |
| hsa04066 HIF-1 signaling pathway | <0.0000 | <0.0000 |
| hsa05206 MicroRNAs in cancer | <0.0000 | <0.0000 |
| hsa05163 Human cytomegalovirus infection | <0.0000 | <0.0000 |
| hsa04550 Signaling pathways regulating pluripotency of stem cells | <0.0000 | <0.0000 |
| hsa01524 Platinum drug resistance | <0.0000 | <0.0000 |
| hsa04068 FoxO signaling pathway | <0.0000 | <0.0000 |
| hsa05220 Chronic myeloid leukemia | <0.0000 | <0.0000 |
| hsa05161 Hepatitis B | <0.0000 | <0.0000 |
| hsa05203 Viral carcinogenesis | <0.0000 | <0.0000 |
| hsa04110 Cell cycle | <0.0000 | <0.0000 |
| hsa05214 Glioma | <0.0000 | <0.0000 |
| hsa05218 Melanoma | <0.0000 | <0.0000 |
| hsa05223 Non-small cell lung cancer | <0.0000 | <0.0000 |
| hsa05200 Pathways in cancer | <0.0000 | <0.0000 |
| hsa05224 Breast cancer | <0.0000 | <0.0000 |
| hsa05160 Hepatitis C | <0.0000 | <0.0000 |
| hsa05213 Endometrial cancer | <0.0000 | <0.0000 |
| hsa05225 Hepatocellular carcinoma | <0.0000 | <0.0000 |
| hsa04012 ErbB signaling pathway | <0.0000 | <0.0000 |
| hsa05210 Colorectal cancer | <0.0000 | <0.0000 |
| hsa05205 Proteoglycans in cancer | <0.0000 | <0.0000 |
| hsa04218 Cellular senescence | <0.0000 | <0.0000 |
| hsa05167 Kaposi sarcoma-associated herpesvirus infection | <0.0000 | <0.0000 |
| hsa01522 Endocrine resistance | <0.0000 | <0.0000 |
| hsa05215 Prostate cancer | <0.0000 | <0.0000 |
| hsa04151 PI3K-Akt signaling pathway | <0.0000 | <0.0000 |
| hsa04115 p53 signaling pathway | <0.0000 | <0.0000 |
| hsa04931 Insulin resistance | 0.0001 | 0.0006 |
| hsa04919 Thyroid hormone signaling pathway | 0.0001 | 0.0006 |
| hsa05211 Renal cell carcinoma | 0.0001 | 0.0006 |
| hsa05169 Epstein-Barr virus infection | 0.0002 | 0.001 |
| hsa05222 Small cell lung cancer | 0.0002 | 0.001 |
| hsa04152 AMPK signaling pathway | 0.0002 | 0.001 |
| hsa04010 MAPK signaling pathway | 0.0002 | 0.001 |
| hsa04933 AGE-RAGE signaling pathway in diabetic complications | 0.0002 | 0.001 |
| hsa04371 Apelin signaling pathway | 0.0002 | 0.001 |
| hsa04630 JAK-STAT signaling pathway | 0.0002 | 0.001 |
| hsa01521 EGFR tyrosine kinase inhibitor resistance | 0.0003 | 0.0014 |
| hsa04917 Prolactin signaling pathway | 0.0003 | 0.0014 |
| hsa04510 Focal adhesion | 0.0003 | 0.0014 |
| hsa04210 Apoptosis | 0.0004 | 0.0017 |
| hsa04211 Longevity regulating pathway | 0.0004 | 0.0017 |
| hsa04934 Cushing syndrome | 0.0004 | 0.0017 |
| hsa05131 Shigellosis | 0.0005 | 0.0021 |
| hsa05226 Gastric cancer | 0.0006 | 0.0024 |
| hsa04910 Insulin signaling pathway | 0.0006 | 0.0024 |
| hsa04150 mTOR signaling pathway | 0.001 | 0.0039 |
| hsa05162 Measles | 0.0012 | 0.0046 |
| hsa04071 Sphingolipid signaling pathway | 0.0012 | 0.0046 |
| hsa04014 Ras signaling pathway | 0.0014 | 0.0052 |
| hsa05230 Central carbon metabolism in cancer | 0.0019 | 0.0069 |
| hsa05216 Thyroid cancer | 0.0021 | 0.0074 |
| hsa04330 Notch signaling pathway | 0.0021 | 0.0074 |
| hsa04213 Longevity regulating pathway | 0.0022 | 0.0076 |
| hsa04520 Adherens junction | 0.0023 | 0.0078 |
| hsa04935 Growth hormone synthesis. secretion and action | 0.0026 | 0.0087 |
| hsa05142 Chagas disease (American trypanosomiasis) | 0.0032 | 0.0105 |
| hsa04144 Endocytosis | 0.0035 | 0.0113 |
| hsa04350 TGF-beta signaling pathway | 0.0036 | 0.0115 |
| hsa05202 Transcriptional misregulation in cancer | 0.0039 | 0.0122 |
| hsa05166 Human T-cell leukemia virus 1 infection | 0.0041 | 0.0125 |
| hsa04141 Protein processing in endoplasmic reticulum | 0.0041 | 0.0125 |
| hsa04928 Parathyroid hormone synthesis. secretion and action | 0.0044 | 0.0132 |
| hsa04390 Hippo signaling pathway | 0.0045 | 0.0133 |
| hsa04530 Tight junction | 0.005 | 0.0144 |
| hsa05170 Human immunodeficiency virus 1 infection | 0.005 | 0.0144 |
| hsa04916 Melanogenesis | 0.0065 | 0.0184 |
| hsa04728 Dopaminergic synapse | 0.0068 | 0.019 |
| hsa04625 C-type lectin receptor signaling pathway | 0.0082 | 0.0226 |
| hsa04062 Chemokine signaling pathway | 0.0085 | 0.0231 |
| hsa05130 Pathogenic Escherichia coli infection | 0.0094 | 0.0252 |
| hsa04720 Long-term potentiation | 0.0097 | 0.0253 |
| hsa05221 Acute myeloid leukemia | 0.0097 | 0.0253 |
| hsa04215 Apoptosis | 0.0101 | 0.0257 |
| hsa04915 Estrogen signaling pathway | 0.01 | 0.0257 |
| hsa04664 Fc epsilon RI signaling pathway | 0.0106 | 0.0266 |
| hsa04926 Relaxin signaling pathway | 0.0111 | 0.0275 |
| hsa04914 Progesterone-mediated oocyte maturation | 0.0119 | 0.0292 |
| hsa04730 Long-term depression | 0.0122 | 0.0295 |
| hsa03015 mRNA surveillance pathway | 0.014 | 0.0335 |
| hsa04725 Cholinergic synapse | 0.0143 | 0.0338 |
| hsa04810 Regulation of actin cytoskeleton | 0.016 | 0.0374 |
| hsa04660 T cell receptor signaling pathway | 0.0169 | 0.0386 |
| hsa04120 Ubiquitin mediated proteolysis | 0.0169 | 0.0386 |
| hsa05164 Influenza A | 0.0178 | 0.0395 |
| hsa04360 Axon guidance | 0.0176 | 0.0395 |
| hsa04140 Autophagy | 0.0179 | 0.0395 |
| hsa04930 Type II diabetes mellitus | 0.0192 | 0.0406 |
| hsa04960 Aldosterone-regulated sodium reabsorption | 0.0192 | 0.0406 |
| hsa04137 Mitophagy | 0.0191 | 0.0406 |
| hsa04310 Wnt signaling pathway | 0.0189 | 0.0406 |
| hsa04380 Osteoclast differentiation | 0.02 | 0.0414 |
| hsa04114 Oocyte meiosis | 0.02 | 0.0414 |
| hsa04659 Th17 cell differentiation | 0.0206 | 0.0423 |
| hsa05231 Choline metabolism in cancer | 0.0228 | 0.0463 |
| hsa05135 Yersinia infection | 0.0236 | 0.0474 |

FDR, false discovery rate corrected p-value.

Supplementary table 3. Identified KEGG pathways of the differentially expressed plasma miRNAs subjected to a target-based pathway enrichment analysis using DAVID.

| Pathway | Fold Enrichment | P Value | FDR |
| --- | --- | --- | --- |
| hsa05200 Pathways in cancer | 2.331375 | 2.21E-10 | 6.08E-08 |
| hsa05214 Glioma | 4.698618 | 5.62E-09 | 5.15E-07 |
| hsa04722 Neurotrophin signaling pathway | 3.514641 | 5.59E-09 | 7.69E-07 |
| hsa05220 Chronic myeloid leukemia | 4.241808 | 3.93E-08 | 2.16E-06 |
| hsa05219 Bladder cancer | 5.67545 | 3.40E-08 | 2.34E-06 |
| hsa05205 Proteoglycans in cancer | 2.690518 | 5.97E-08 | 2.74E-06 |
| hsa04068 FoxO signaling pathway | 3.147439 | 7.29E-08 | 2.86E-06 |
| hsa05223 Non-small cell lung cancer | 4.674645 | 9.97E-08 | 3.05E-06 |
| hsa05210 Colorectal cancer | 4.45683 | 9.00E-08 | 3.09E-06 |
| hsa04066 HIF-1 signaling pathway | 3.484342 | 3.49E-07 | 9.60E-06 |
| hsa05218 Melanoma | 3.89188 | 8.47E-07 | 2.12E-05 |
| hsa05212 Pancreatic cancer | 4.027387 | 1.06E-06 | 2.25E-05 |
| hsa05203 Viral carcinogenesis | 2.483009 | 1.04E-06 | 2.39E-05 |
| hsa05161 Hepatitis B | 2.808369 | 1.42E-06 | 2.79E-05 |
| hsa04115 p53 signaling pathway | 3.907166 | 1.69E-06 | 3.10E-05 |
| hsa04550 Signaling pathways regulating pluripotency of stem cells | 2.804787 | 2.35E-06 | 4.04E-05 |
| hsa04012 ErbB signaling pathway | 3.343297 | 4.76E-06 | 7.71E-05 |
| hsa05215 Prostate cancer | 3.305305 | 5.70E-06 | 8.70E-05 |
| hsa04151 PI3K-Akt signaling pathway | 1.981267 | 7.72E-06 | 1.12E-04 |
| hsa05160 Hepatitis C | 2.733711 | 9.66E-06 | 1.26E-04 |
| hsa04110 Cell cycle | 2.81484 | 9.23E-06 | 1.27E-04 |
| hsa04919 Thyroid hormone signaling pathway | 2.782204 | 2.89E-05 | 3.61E-04 |
| hsa05213 Endometrial cancer | 3.915515 | 3.38E-05 | 4.04E-04 |
| hsa04931 Insulin resistance | 2.827872 | 3.62E-05 | 4.15E-04 |
| hsa05211 Renal cell carcinoma | 3.305305 | 1.19E-04 | 0.001259 |
| hsa05206 MicroRNAs in cancer | 1.932332 | 1.18E-04 | 0.001298 |
| hsa04152 AMPK signaling pathway | 2.483009 | 2.37E-04 | 0.002323 |
| hsa04010 MAPK signaling pathway | 1.954441 | 2.36E-04 | 0.002404 |
| hsa04520 Adherens junction | 3.072537 | 2.70E-04 | 0.002554 |
| hsa04510 Focal adhesion | 2.047363 | 3.53E-04 | 0.003236 |
| hsa04910 Insulin signaling pathway | 2.318504 | 4.25E-04 | 0.003764 |
| hsa04917 Prolactin signaling pathway | 2.867701 | 9.30E-04 | 0.007963 |
| hsa05230 Central carbon metabolism in cancer | 2.954116 | 0.001167 | 0.009686 |
| hsa04071 Sphingolipid signaling pathway | 2.302696 | 0.001276 | 0.010273 |
| hsa05142 Chagas disease (American trypanosomiasis) | 2.377277 | 0.001785 | 0.013553 |
| hsa05222 Small cell lung cancer | 2.566472 | 0.001756 | 0.013713 |
| hsa04144 Endocytosis | 1.810374 | 0.00202 | 0.014524 |
| hsa04630 Jak-STAT signaling pathway | 2.106277 | 0.002011 | 0.014854 |
| hsa05166 HTLV-I infection | 1.774975 | 0.002283 | 0.015989 |
| hsa04014 Ras signaling pathway | 1.80183 | 0.003156 | 0.0215 |
| hsa04390 Hippo signaling pathway | 2.022584 | 0.003265 | 0.021694 |
| hsa05221 Acute myeloid leukemia | 2.856728 | 0.004275 | 0.027662 |
| hsa04350 TGF-beta signaling pathway | 2.42389 | 0.00443 | 0.027994 |
| hsa04915 Estrogen signaling pathway | 2.203536 | 0.007284 | 0.044661 |
| hsa04916 Melanogenesis | 2.181501 | 0.007955 | 0.047636 |
| hsa04062 Chemokine signaling pathway | 1.79837 | 0.008245 | 0.048292 |
| hsa04114 Oocyte meiosis | 2.096337 | 0.008478 | 0.048595 |
| hsa04320 Dorso-ventral axis formation | 3.770496 | 0.008668 | 0.048656 |

FDR, false discovery rate corrected p-value.

Supplementary table 4. Identified KEGG pathways of the differentially expressed liver miRNAs subjected to a target-based pathway enrichment analysis using miRWalk 3.0.

| Pathway | P value | FDR |
| --- | --- | --- |
| hsa05219 Bladder cancer | <0.0000 | <0.0000 |
| hsa04152 AMPK signaling pathway | <0.0000 | <0.0000 |
| hsa04722 Neurotrophin signaling pathway | <0.0000 | <0.0000 |
| hsa04210 Apoptosis | <0.0000 | <0.0000 |
| hsa05212 Pancreatic cancer | <0.0000 | <0.0000 |
| hsa01521 EGFR tyrosine kinase inhibitor resistance | <0.0000 | <0.0000 |
| hsa05163 Human cytomegalovirus infection | <0.0000 | <0.0000 |
| hsa01524 Platinum drug resistance | <0.0000 | <0.0000 |
| hsa05220 Chronic myeloid leukemia | <0.0000 | <0.0000 |
| hsa04510 Focal adhesion | <0.0000 | <0.0000 |
| hsa05214 Glioma | <0.0000 | <0.0000 |
| hsa05218 Melanoma | <0.0000 | <0.0000 |
| hsa05223 Non-small cell lung cancer | <0.0000 | <0.0000 |
| hsa05200 Pathways in cancer | <0.0000 | <0.0000 |
| hsa05224 Breast cancer | <0.0000 | <0.0000 |
| hsa05160 Hepatitis C | <0.0000 | <0.0000 |
| hsa05213 Endometrial cancer | <0.0000 | <0.0000 |
| hsa05210 Colorectal cancer | <0.0000 | <0.0000 |
| hsa05205 Proteoglycans in cancer | <0.0000 | <0.0000 |
| hsa05167 Kaposi sarcoma-associated herpesvirus infection | <0.0000 | <0.0000 |
| hsa01522 Endocrine resistance | <0.0000 | <0.0000 |
| hsa05215 Prostate cancer | <0.0000 | <0.0000 |
| hsa04151 PI3K-Akt signaling pathway | <0.0000 | <0.0000 |
| hsa04115 p53 signaling pathway | <0.0000 | <0.0000 |
| hsa05222 Small cell lung cancer | 0.0001 | 0.0006 |
| hsa04211 Longevity regulating pathway | 0.0001 | 0.0006 |
| hsa04919 Thyroid hormone signaling pathway | 0.0001 | 0.0006 |
| hsa05230 Central carbon metabolism in cancer | 0.0001 | 0.0006 |
| hsa04140 Autophagy | 0.0001 | 0.0006 |
| hsa05206 MicroRNAs in cancer | 0.0002 | 0.0011 |
| hsa04068 FoxO signaling pathway | 0.0002 | 0.0011 |
| hsa05162 Measles | 0.0002 | 0.0011 |
| hsa05225 Hepatocellular carcinoma | 0.0002 | 0.0011 |
| hsa04725 Cholinergic synapse | 0.0003 | 0.0016 |
| hsa05226 Gastric cancer | 0.0004 | 0.0019 |
| hsa04215 Apoptosis | 0.0004 | 0.0019 |
| hsa04340 Hedgehog signaling pathway | 0.0004 | 0.0019 |
| hsa05211 Renal cell carcinoma | 0.0004 | 0.0019 |
| hsa04213 Longevity regulating pathway | 0.0005 | 0.0022 |
| hsa04550 Signaling pathways regulating pluripotency of stem cells | 0.0005 | 0.0022 |
| hsa04931 Insulin resistance | 0.0006 | 0.0026 |
| hsa04066 HIF-1 signaling pathway | 0.0007 | 0.003 |
| hsa04218 Cellular senescence | 0.0008 | 0.0033 |
| hsa05161 Hepatitis B | 0.0009 | 0.0035 |
| hsa04110 Cell cycle | 0.0009 | 0.0035 |
| hsa04630 JAK-STAT signaling pathway | 0.0009 | 0.0035 |
| hsa05165 Human papillomavirus infection | 0.001 | 0.0037 |
| hsa05216 Thyroid cancer | 0.001 | 0.0037 |
| hsa05166 Human T-cell leukemia virus 1 infection | 0.0011 | 0.0039 |
| hsa04910 Insulin signaling pathway | 0.0011 | 0.0039 |
| hsa04150 mTOR signaling pathway | 0.0013 | 0.0045 |
| hsa04917 Prolactin signaling pathway | 0.0013 | 0.0045 |
| hsa04015 Rap1 signaling pathway | 0.0014 | 0.0047 |
| hsa04710 Circadian rhythm | 0.0016 | 0.0053 |
| hsa04144 Endocytosis | 0.0025 | 0.008 |
| hsa04137 Mitophagy | 0.0025 | 0.008 |
| hsa05168 Herpes simplex virus 1 infection | 0.0026 | 0.0082 |
| hsa04921 Oxytocin signaling pathway | 0.0027 | 0.0083 |
| hsa04010 MAPK signaling pathway | 0.0028 | 0.0085 |
| hsa04934 Cushing syndrome | 0.003 | 0.009 |
| hsa05170 Human immunodeficiency virus 1 infection | 0.0033 | 0.0097 |
| hsa05169 Epstein-Barr virus infection | 0.0039 | 0.0111 |
| hsa05203 Viral carcinogenesis | 0.0039 | 0.0111 |
| hsa04014 Ras signaling pathway | 0.0043 | 0.012 |
| hsa04713 Circadian entrainment | 0.0049 | 0.0135 |
| hsa04012 ErbB signaling pathway | 0.0051 | 0.0138 |
| hsa05418 Fluid shear stress and atherosclerosis | 0.0057 | 0.0152 |
| hsa04728 Dopaminergic synapse | 0.0085 | 0.0224 |
| hsa04962 Vasopressin-regulated water reabsorption | 0.0089 | 0.0228 |
| hsa04071 Sphingolipid signaling pathway | 0.0088 | 0.0228 |
| hsa04371 Apelin signaling pathway | 0.0112 | 0.0282 |
| hsa04915 Estrogen signaling pathway | 0.0118 | 0.0289 |
| hsa04611 Platelet activation | 0.0118 | 0.0289 |
| hsa05017 Spinocerebellar ataxia | 0.0127 | 0.0307 |
| hsa04933 AGE-RAGE signaling pathway in diabetic complications | 0.0144 | 0.0344 |
| hsa04926 Relaxin signaling pathway | 0.0155 | 0.036 |
| hsa04916 Melanogenesis | 0.0153 | 0.036 |
| hsa04062 Chemokine signaling pathway | 0.0167 | 0.0383 |
| hsa04932 Non-alcoholic fatty liver disease (NAFLD) | 0.0204 | 0.0462 |
| hsa04912 GnRH signaling pathway | 0.0216 | 0.0483 |

FDR, false discovery rate corrected p-value.

Supplementary table 5. Identified KEGG pathways of the differentially expressed liver miRNAs subjected to a target-based pathway enrichment analysis using DAVID.

| Pathway | Fold Enrichment | P Value | FDR |
| --- | --- | --- | --- |
| hsa05212 Pancreatic cancer | 5.354533 | 5.13E-08 | 6.24E-06 |
| hsa05223 Non-small cell lung cancer | 5.84949 | 3.96E-08 | 9.62E-06 |
| hsa05215 Prostate cancer | 4.420353 | 1.61E-07 | 1.30E-05 |
| hsa04151 PI3K-Akt signaling pathway | 2.373706 | 5.38E-07 | 3.27E-05 |
| hsa04152 AMPK signaling pathway | 3.495427 | 1.63E-06 | 7.91E-05 |
| hsa04510 Focal adhesion | 2.782767 | 2.06E-06 | 8.36E-05 |
| hsa05219 Bladder cancer | 5.99216 | 2.63E-06 | 9.12E-05 |
| hsa05205 Proteoglycans in cancer | 2.763884 | 3.75E-06 | 1.14E-04 |
| hsa04722 Neurotrophin signaling pathway | 3.412202 | 4.47E-06 | 1.21E-04 |
| hsa05213 Endometrial cancer | 5.118304 | 5.17E-06 | 1.26E-04 |
| hsa05210 Colorectal cancer | 4.622984 | 6.53E-06 | 1.44E-04 |
| hsa05220 Chronic myeloid leukemia | 4.265253 | 7.34E-06 | 1.49E-04 |
| hsa05214 Glioma | 4.409615 | 1.13E-05 | 2.11E-04 |
| hsa05200 Pathways in cancer | 2.083788 | 1.35E-05 | 2.35E-04 |
| hsa05166 HTLV-I infection | 2.337493 | 3.76E-05 | 5.71E-04 |
| hsa04919 Thyroid hormone signaling pathway | 3.204503 | 3.60E-05 | 5.82E-04 |
| hsa05230 Central carbon metabolism in cancer | 4.158622 | 4.85E-05 | 6.94E-04 |
| hsa05161 Hepatitis B | 2.823892 | 7.00E-05 | 9.44E-04 |
| hsa05218 Melanoma | 3.748617 | 1.39E-04 | 0.001778 |
| hsa05222 Small cell lung cancer | 3.372059 | 2.11E-04 | 0.002555 |
| hsa04068 FoxO signaling pathway | 2.750133 | 2.50E-04 | 0.002887 |
| hsa05211 Renal cell carcinoma | 3.722403 | 2.99E-04 | 0.003024 |
| hsa05206 MicroRNAs in cancer | 2.075955 | 2.99E-04 | 0.003151 |
| hsa04725 Cholinergic synapse | 2.951094 | 2.87E-04 | 0.00317 |
| hsa04115 p53 signaling pathway | 3.666844 | 3.43E-04 | 0.003324 |
| hsa04550 Signaling pathways regulating pluripotency of stem cells | 2.63227 | 4.22E-04 | 0.003941 |
| hsa04066 HIF-1 signaling pathway | 2.985677 | 7.09E-04 | 0.005928 |
| hsa04931 Insulin resistance | 2.843502 | 6.99E-04 | 0.006051 |
| hsa05160 Hepatitis C | 2.616877 | 6.88E-04 | 0.006179 |
| hsa04110 Cell cycle | 2.641705 | 9.49E-04 | 0.007663 |
| hsa04910 Insulin signaling pathway | 2.522063 | 0.001032 | 0.008059 |
| hsa04015 Rap1 signaling pathway | 2.144813 | 0.001288 | 0.009739 |
| hsa04144 Endocytosis | 2.038826 | 0.001454 | 0.010657 |
| hsa05216 Thyroid cancer | 4.94181 | 0.002337 | 0.016581 |
| hsa04921 Oxytocin signaling pathway | 2.320298 | 0.002484 | 0.017121 |
| hsa04210 Apoptosis | 3.302131 | 0.002855 | 0.019114 |
| hsa04710 Circadian rhythm | 4.622984 | 0.003336 | 0.02171 |
| hsa05203 Viral carcinogenesis | 1.997387 | 0.00506 | 0.031919 |
| hsa04713 Circadian entrainment | 2.58609 | 0.00618 | 0.037888 |
| hsa04014 Ras signaling pathway | 1.902378 | 0.006837 | 0.040822 |
| hsa04917 Prolactin signaling pathway | 2.883551 | 0.007146 | 0.041612 |
| hsa04915 Estrogen signaling pathway | 2.481602 | 0.008397 | 0.047616 |

FDR, false discovery rate corrected p-value.

**
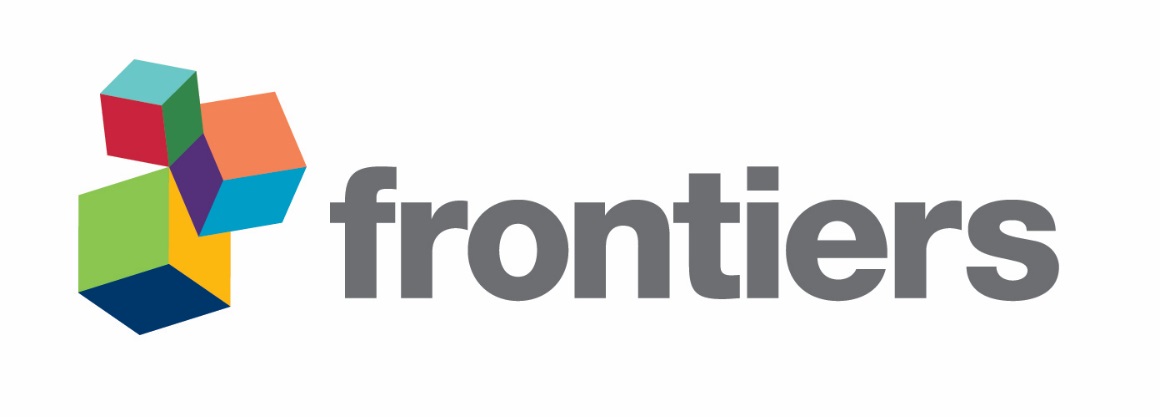
**
